# Supplementary material for: In silico modeling human VPS13 proteins associated with donor and target membranes suggests lipid transfer mechanisms
Source: Proteins. 2022 Dec 5;91(4):439–55. doi: 10.1002/prot.26446 (PMC10953354; doi:10.1002/prot.26446)
Supplement: Supplementary file 1 — Appendix S1: Supporting Information [file PROT-91-439-s002.docx]

**Supplementary material**

Table S1. BackPhyre (curated) results for the submission of the VPS13A small globular domain against the Homo Sapiens genome database (Kelley et al, 2015).

| **Hit** | **Alignment coverage** | **Confidence** | **Percentage identity** |
| --- | --- | --- | --- |
| E3 ubiquitin-protein ligase DTX1 (NP_004407.2) | 1-68 | 98.3 | 20 |
| Deltex 2 (NP_065943.1) | 1-68 | 98.1 | 20 |
| SEC23-interacting protein (NP_009121.1) | 4-63 | 95.6 | 24 |
| DDHD domain containing 1 (NP_085140.1) | 4-50 | 92.7 | 17 |
| DDHD domain containing 2 (NP_056029.1) | 4-63 | 92.0 | 17 |

Table S2. BackPhyre (curated) results for the submission of the VPS13B small globular domain against the Homo Sapiens genome database (Kelley et al, 2015).

| **Hit** | **Alignment coverage** | **Confidence** | **Percentage identity** |
| --- | --- | --- | --- |
| N/A | N/A | N/A | N/A |

Table S3. BackPhyre (curated) results for the submission of the VPS13C small globular domain against the Homo Sapiens genome database (Kelley et al, 2015).

| **Hit** | **Alignment coverage** | **Confidence** | **Percentage identity** |
| --- | --- | --- | --- |
| Methanethiol oxidase (NP_003935.2) | 14-38 | 53.9 | 44 |

Table S4. BackPhyre (curated) results for the submission of the VPS13D small globular domain against the Homo Sapiens genome database (Kelley et al, 2015).

| **Hit** | **Alignment coverage** | **Confidence** | **Percentage identity** |
| --- | --- | --- | --- |
| GalNAc GALT-10 (NP_060010.3) | 28-126 | 97.7 | 27 |
| GalNAc-T12 (NP_078918.2) | 32-165 | 97.5 | 19 |
| Absent in melanoma 1-like protein (NP_001034864.1) | 18-126 | 97.5 | 20 |
| GALT-9 (NP_068580.2) | 32-144 | 97.5 | 22 |
| Beta/gamma crystallin domain-containing protein 3 (NP_705833.2) | 18-126 | 97.4 | 24 |
| GALT-17 (NP_001030017.1) | 28-127 | 97.2 | 27 |
| GALT-17 (NP_071924.1) | 32-156 | 97.1 | 20 |
| GALT-10 (NP_938080.1) | 28-126 | 97.0 | 26 |
| GALT-13 (NP_443149.1) | 23-127 | 96.9 | 31 |
| GALT-1 (NP_065207.2) | 23-126 | 96.8 | 39 |
| GALT-4 (NP_003765.2) | 32-178 | 96.4 | 14 |
| UDP-N-acetyl-alpha-D-galactosamine:GALT-2 (NP_473451.2) | 29-127 | 96.4 | 29 |
| GALT-2 (NP_004472.1) | 23-126 | 96.0 | 27` |
| GALT-14 (NP_078848.2) | 23-126 | 96.0 | 26 |
| GALNAC-T11 (NP_071370.1) | 32-165 | 95.2 | 21 |
| GALT-6 (NP_009141.1) | 32-143 | 95.2 | 12 |
| GALT-8 (NP_059113.1) | 26-126 | 94.9 | 19 |
| GALT-7 (NP_059119.1) | 23-126 | 94.8 | 19 |
| UDP-N-acetyl-alpha-D-galactosamine:GALT-4 (NP_940918.1) | 32-144 | 94.4 | 15 |
| GALT-5 (NP_055383.1) | 23-127 | 94.2 | 17 |
| GALT-3 (NP_004473.1) | 32-143 | 93.9 | 20 |
| PLA2R (NP_001007268.1) | 1-177 | 84.6 | 16 |
| GALT-1 (NP_065743.1) | 28-125 | 80.4 | 33 |
| PLA2R (NP_031392.3) | 1-177 | 78.7 | 16 |
| MRC1 (NP_002429.1) | 35-177 | 61.8 | 15 |

Table S5. BackPhyre (curated) results for the submission of the VPS13A gondola domain against the Homo Sapiens genome database (Kelley et al, 2015).

| **Hit** | **Alignment coverage** | **Confidence** | **Percentage identity** |
| --- | --- | --- | --- |
| ATG2A (NP_055919.1) | 2-191 | 100.0 | 18 |
| MTCH1 (NP_055156.1) | 28-170 | 75.1 | 24 |
| Plin-2 (NP_001113.2) | 24-188 | 62.3 | 11 |
| MPCPB (NP_998776.1) | 28-178 | 51.0 | 18 |

Table S6. BackPhyre (curated) results for the submission of the VPS13B gondola domain against the Homo Sapiens genome database (Kelley et al, 2015).

| **Hit** | **Alignment coverage** | **Confidence** | **Percentage identity** |
| --- | --- | --- | --- |
| ATG2A (NP_055919.1) | 2-200 | 100.0 | 19 |
| MTCH1 (NP_055156.1) | 28-179 | 56.8 | 15 |

Table S7. BackPhyre (curated) results for the submission of the VPS13C gondola domain against the Homo Sapiens genome database (Kelley et al, 2015).

| **Hit** | **Alignment coverage** | **Confidence** | **Percentage identity** |
| --- | --- | --- | --- |
| ATG2A (NP_055919.1) | 2-193 | 100.0 | 19 |

Table S8. BackPhyre (curated) results for the submission of the VPS13D gondola domain against the Homo Sapiens genome database (Kelley et al, 2015).

| **Hit** | **Alignment coverage** | **Confidence** | **Percentage identity** |
| --- | --- | --- | --- |
| ATG2A (NP_055919.1) | 2-174 | 99.9 | 18 |
| MTCH1 (NP_055156.1) | 29-153 | 73.2 | 16 |
| MTCH2 (NP_055157.1) | 29-169 | 64.8 | 13 |

Table S9. Distribution and functional significance of pathogenic missense mutations in human VPS13 proteins (National Center for Biotechnology Information, ClinVar; https://www.ncbi.nlm.nih.gov/clinvar, accessed July 24, 2022). The nomenclature may vary from the ClinVar database reports as it was adapted to the canonical transcripts used for modelling.

| **VPS13A (NP_150648.2)** | | | |
| --- | --- | --- | --- |
| **Nomenclature** | **Location** | **Topological orientation** | **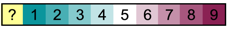Conservation (0-9)** |
| [p.Ile90Lys](https://www.ncbi.nlm.nih.gov/clinvar/variation/4682/) | Chorein β-sheet | Groove inward-facing | 6 |
| [p.Ala2679Thr](https://www.ncbi.nlm.nih.gov/clinvar/variation/4686/) | β-sheet between SGD-Gondola | Cytosolic-facing | 6 |
| **VPS13B (NP_060360.3)** | | | |
| [p.Met1Ile](https://www.ncbi.nlm.nih.gov/clinvar/variation/402220/) | Pre-Chorein loop | Cytosolic-facing | 9 |
| [p.Gln27Arg](https://www.ncbi.nlm.nih.gov/clinvar/variation/813329/) | Chorein β-sheet | Cytosolic-facing | 9 |
| [p.Pro1133Ser](https://www.ncbi.nlm.nih.gov/clinvar/variation/225021/) | RBG loop | Cytosolic-facing | 6 |
| [p.Ile1636Asn](https://www.ncbi.nlm.nih.gov/clinvar/variation/977843/) | RBG β-sheet | Inward-facing | 9 |
| [p.Ser1812Ile](https://www.ncbi.nlm.nih.gov/clinvar/variation/427100/) | RBG loop | Cytosolic-facing | 6 |
| [p.Leu2193Arg](https://www.ncbi.nlm.nih.gov/clinvar/variation/2819/) | RBG β-sheet | Inward-facing | 4 |
| [p.Leu2543Val](https://www.ncbi.nlm.nih.gov/clinvar/variation/975775/) | RBG β-sheet | Cytosolic/SGD-facing | 2 |
| **VPS13C (NP_065872.1)** | | | |
| [p.Gly1389Arg](https://www.ncbi.nlm.nih.gov/clinvar/variation/222070/) | RBG loop | Cytosolic-facing | 1 |
| **VPS13D (NP_056193.2)** | | | |
| [p.Ile1170Ser](https://www.ncbi.nlm.nih.gov/clinvar/variation/806071/) | RBG β-sheet | Inward-facing | 9 |
| [p.Gly1190Asp](https://www.ncbi.nlm.nih.gov/clinvar/variation/561198/) | RBG loop | Cytosolic-facing | 6 |
| [p.Asn3521Ser](https://www.ncbi.nlm.nih.gov/clinvar/variation/561203/) | VAB domain β-sheet | Cytosolic-facing | 6 |
| [p.Ala4139Val](https://www.ncbi.nlm.nih.gov/clinvar/variation/932894/) | Gondola domain H4 | Membrane-bound | 8 |
| [p.Gly4177Asp](https://www.ncbi.nlm.nih.gov/clinvar/variation/983263/) | Gondola domain hinge between H5-H6 | Membrane-bound | 9 |
| [p.Ala4210Val](https://www.ncbi.nlm.nih.gov/clinvar/variation/561200/) | Gondola domain H6 | Membrane-bound | 9 |
| [p.Arg4228Gln](https://www.ncbi.nlm.nih.gov/clinvar/variation/561204/) | Loop between gondola and PH-like domains | Cytosolic-facing | 6 |
| [p.Ala4248Glu](https://www.ncbi.nlm.nih.gov/clinvar/variation/807716/) | PH-like domain α-helix | Cytosolic/VAB-facing | 9 |

Table S10. Protein-membrane system calculations between the N-terminus (1-215 aa) of VPS13A and an ER membrane (Brooks et al, 2009; Jo et al, 2008, Wu et al, 2014).

| **Calculated Lipid Number** | | |
| --- | --- | --- |
| **Lipid type** | **Upper-leaflet number** | **Lower-leaflet number** |
| Cholesterol | 15 | 15 |
| POPA | 3 | 3 |
| POPC | 48 | 48 |
| PLPC | 54 | 54 |
| PSPE | 24 | 24 |
| POPE | 15 | 15 |
| OLPS | 9 | 9 |
| SLPI | 9 | 9 |
| SAPI | 9 | 9 |
| SAPC | 81 | 81 |
| SAPE | 21 | 21 |
| PSM | 12 | 12 |
| **Calculated XY system size** | | |
|  | **Upper-leaflet** | **Lower-leaflet** |
| Protein Area | 0 | 657.992985 |
| Lipid area | 19369.8 | 19369.8 |
| Number of Lipids | 300 | 300 |
| Total area | 19369.8 | 20027.792985 |
| Protein X extent | 33.42 | |
| Protein Y extent | 31.86 | |
| Average area | 19698.80 | |
| A | 140.35 | |
| B | 140.35 | |

Table S11. Protein-membrane system calculations between the C-terminus (1876-3174 aa) of VPS13A and an outer mitochondrial membrane (Brooks et al, 2009; Jo et al, 2008, Wu et al, 2014).

| **Calculated Lipid Number** | | |
| --- | --- | --- |
| **Lipid type** | **Upper-leaflet number** | **Lower-leaflet number** |
| POPA | 3 | 3 |
| POPC | 18 | 18 |
| PLPC | 54 | 54 |
| POPE | 6 | 12 |
| PLPE | 15 | 27 |
| SAPI | 66 | 12 |
| TLCL1 | 0 | 6 |
| SAPC | 90 | 90 |
| SAPE | 39 | 75 |
| SAPS | 9 | 3 |
| **Calculated XY system size** | | |
|  | **Upper-leaflet** | **Lower-leaflet** |
| Protein Area | 0 | 1798.08463 |
| Lipid area | 20153.1 | 20317.5 |
| Number of Lipids | 300 | 300 |
| Total area | 20153.1 | 22115.58463 |
| Protein X extent | 71.44 | |
| Protein Y extent | 58.24 | |
| Average area | 21134.34 | |
| A | 145.38 | |
| B | 145.38 | |

Table S12. Protein-membrane system calculations between the N-terminus (1-205 aa) of VPS13B and a Golgi complex membrane (Brooks et al, 2009; Jo et al, 2008, Wu et al, 2014).

| **Calculated Lipid Number** | | |
| --- | --- | --- |
| **Lipid type** | **Upper-leaflet number** | **Lower-leaflet number** |
| Cholesterol | 24 | 24 |
| POPC | 33 | 33 |
| PLPC | 42 | 42 |
| PSPE | 24 | 24 |
| PLPE | 12 | 12 |
| POPS | 12 | 12 |
| PSPI | 6 | 6 |
| POPI | 15 | 15 |
| SAPI | 6 | 6 |
| SAPC | 60 | 60 |
| SAPE | 15 | 15 |
| TSM | 36 | 36 |
| LPC16 | 15 | 15 |
| **Calculated XY system size** | | |
|  | **Upper-leaflet** | **Lower-leaflet** |
| Protein Area | 0 | 989.62037 |
| Lipid area | 18298.2 | 18298.2 |
| Number of Lipids | 300 | 300 |
| Total area | 18298.2 | 19287.82037 |
| Protein X extent | 45.07 | |
| Protein Y extent | 22.33 | |
| Average area | 18793.01 | |
| A | 137.09 | |
| B | 137.09 | |

Table S13. Protein-membrane system calculations between the C-terminus (2630-4022 aa) of VPS13B and an outer mitochondrial membrane (Brooks et al, 2009; Jo et al, 2008, Wu et al, 2014).

| **Calculated Lipid Number** | | |
| --- | --- | --- |
| **Lipid type** | **Upper-leaflet number** | **Lower-leaflet number** |
| POPA | 3 | 3 |
| POPC | 18 | 18 |
| PLPC | 54 | 54 |
| POPE | 6 | 12 |
| PLPE | 15 | 27 |
| SAPI | 66 | 12 |
| TLCL1 | 0 | 6 |
| SAPC | 90 | 90 |
| SAPE | 39 | 75 |
| SAPS | 9 | 3 |
| **Calculated XY system size** | | |
|  | **Upper-leaflet** | **Lower-leaflet** |
| Protein Area | 0 | 2319.90896 |
| Lipid area | 20153.1 | 20317.5 |
| Number of Lipids | 300 | 300 |
| Total area | 20153.1 | 22637.40896 |
| Protein X extent | 63.27 | |
| Protein Y extent | 84.56 | |
| Average area | 21395.25 | |
| A | 146.27 | |
| B | 146.27 | |

Table S14. Protein-membrane system calculations between the N-terminus (1-225 aa) of VPS13C and an ER membrane (Brooks et al, 2009; Jo et al, 2008, Wu et al, 2014).

| **Calculated Lipid Number** | | |
| --- | --- | --- |
| **Lipid type** | **Upper-leaflet number** | **Lower-leaflet number** |
| Cholesterol | 15 | 15 |
| POPA | 3 | 3 |
| POPC | 48 | 48 |
| PLPC | 54 | 54 |
| PSPE | 24 | 24 |
| POPE | 15 | 15 |
| OLPS | 9 | 9 |
| SLPI | 9 | 9 |
| SAPI | 9 | 9 |
| SAPC | 81 | 81 |
| SAPE | 21 | 21 |
| PSM | 12 | 12 |
| **Calculated XY system size** | | |
|  | **Upper-leaflet** | **Lower-leaflet** |
| Protein Area | 0 | 630.23516 |
| Lipid area | 19369.8 | 19369.8 |
| Number of Lipids | 300 | 300 |
| Total area | 19369.8 | 20000.03516 |
| Protein X extent | 52.74 | |
| Protein Y extent | 24.90 | |
| Average area | 19684.92 | |
| A | 140.30 | |
| B | 140.30 | |

Table S15. Protein-membrane system calculations between the C-terminus (2444-3783 aa) of VPS13C and an endosomal membrane (Brooks et al, 2009; Jo et al, 2008, Wu et al, 2014).

| **Calculated Lipid Number** | | |
| --- | --- | --- |
| **Lipid type** | **Upper-leaflet number** | **Lower-leaflet number** |
| PLPC | 10 | 10 |
| SAPC | 16 | 16 |
| SDPC | 6 | 6 |
| PLPE | 2 | 2 |
| PDoPE | 4 | 4 |
| SAPE | 6 | 6 |
| SAPI | 6 | 6 |
| SDPI | 2 | 2 |
| DSM | 8 | 8 |
| OSM | 8 | 8 |
| Cholesterol | 32 | 32 |
| BMGP | 280 | 280 |
| **Calculated XY system size** | | |
|  | **Upper-leaflet** | **Lower-leaflet** |
| Protein Area | 0 | 489.54831 |
| Lipid area | 23849 | 23849 |
| Number of Lipids | 380 | 380 |
| Total area | 23849 | 24338.54831 |
| Protein X extent | 68.14 | |
| Protein Y extent | 54.98 | |
| Average area | 24093.77 | |
| A | 155.22 | |
| B | 155.22 | |

Table S16. Protein-membrane system calculations between the C-terminus (2444-3783 aa) of VPS13C and an outer mitochondrial membrane (Brooks et al, 2009; Jo et al, 2008, Wu et al, 2014).

| **Calculated Lipid Number** | | |
| --- | --- | --- |
| **Lipid type** | **Upper-leaflet number** | **Lower-leaflet number** |
| POPA | 4 | 4 |
| POPC | 24 | 24 |
| PLPC | 72 | 72 |
| POPE | 8 | 16 |
| PLPE | 20 | 36 |
| SAPI | 88 | 16 |
| TLCL1 | 0 | 8 |
| SAPC | 120 | 120 |
| SAPE | 52 | 100 |
| SAPS | 12 | 4 |
| **Calculated XY system size** | | |
|  | **Upper-leaflet** | **Lower-leaflet** |
| Protein Area | 0 | 489.54831 |
| Lipid area | 23984.8 | 23984.8 |
| Number of Lipids | 382 | 382 |
| Total area | 23984.8 | 24474.34831 |
| Protein X extent | 68.14 | |
| Protein Y extent | 54.98 | |
| Average area | 24229.57 | |
| A | 155.66 | |
| B | 155.66 | |

Table S17. Protein-membrane system calculations between the N-terminus (1-225 aa) of VPS13D and a lipid droplet monolayer (Brooks et al, 2009; Jo et al, 2008, Wu et al, 2014).

| **Calculated Lipid Number** | | |
| --- | --- | --- |
| **Lipid type** | **Upper-leaflet number** | **Lower-leaflet number** |
| Cholesterol | 15 | 15 |
| POPA | 3 | 3 |
| POPC | 48 | 48 |
| PLPC | 54 | 54 |
| PSPE | 24 | 24 |
| POPE | 15 | 15 |
| OLPS | 9 | 9 |
| SLPI | 9 | 9 |
| SAPI | 9 | 9 |
| SAPC | 81 | 81 |
| SAPE | 21 | 21 |
| PSM | 12 | 12 |
| **Calculated XY system size** | | |
|  | **Upper-leaflet** | **Lower-leaflet** |
| Protein Area | 0 | 1049.68063 |
| Lipid area | 19369.8 | 19369.8 |
| Number of Lipids | 300 | 300 |
| Total area | 19369.8 | 20419.48063 |
| Protein X extent | 33.48 | |
| Protein Y extent | 30.34 | |
| Average area | 19894.64 | |
| A | 141.05 | |
| B | 141.05 | |

Table S18. Protein-membrane system calculations between the C-terminus (2889-4388 aa) of VPS13D and an outer mitochondrial membrane (Brooks et al, 2009; Jo et al, 2008, Wu et al, 2014).

| **Calculated Lipid Number** | | |
| --- | --- | --- |
| **Lipid type** | **Upper-leaflet number** | **Lower-leaflet number** |
| POPA | 3 | 3 |
| POPC | 18 | 18 |
| PLPC | 54 | 54 |
| POPE | 6 | 12 |
| PLPE | 15 | 27 |
| SAPI | 66 | 12 |
| TLCL1 | 0 | 6 |
| SAPC | 90 | 90 |
| SAPE | 39 | 75 |
| SAPS | 9 | 3 |
| **Calculated XY system size** | | |
|  | **Upper-leaflet** | **Lower-leaflet** |
| Protein Area | 0 | 1673.81361 |
| Lipid area | 20153.1 | 20317.5 |
| Number of Lipids | 300 | 300 |
| Total area | 20153.1 | 21991.31361 |
| Protein X extent | 97.00 | |
| Protein Y extent | 57.57 | |
| Average area | 21072.21 | |
| A | 145.16 | |
| B | 145.16 | |
